# Health behavior of Austrian tertiary students focusing on diet type linked to sports and exercise—first glimpse of results from the “sustainably healthy—from science 2 high school and university” study

**DOI:** 10.3389/fpubh.2023.1129004

**Published:** 2023-07-18

**Authors:** Katharina C. Wirnitzer, Mohamad Motevalli, Armando Cocca, Derrick R. Tanous, Gerold Wirnitzer, Karl-Heinz Wagner, Manuel Schätzer, Clemens Drenowatz, Gerhard Ruedl, Werner Kirschner

**Affiliations:** ^1^Department of Research and Development in Teacher Education, University College of Teacher Education Tyrol, Innsbruck, Austria; ^2^Department of Sport Science, University of Innsbruck, Innsbruck, Austria; ^3^Research Center Medical Humanities, University of Innsbruck, Innsbruck, Austria; ^4^adventureV & change2V, Stans, Austria; ^5^Department of Nutritional Sciences and Research Platform Active Ageing, University of Vienna, Vienna, Austria; ^6^Special Institute for Preventive Cardiology and Nutrition (SIPCAN), Elsbethen, Austria; ^7^Division of Sport, Physical Activity and Health, University of Teacher Education Upper Austria, Linz, Austria

**Keywords:** vegan, vegetarian, plant-based, physical activity, obesity, public health, education, sustainable

## Abstract

**Background:**

There is a strong association between lifestyle behavior and health status. While young adulthood is a critical period for adopting and stabilizing lifelong healthy behavior, university life is independently associated with psychological stressors that may further affect health and well-being.

**Objective:**

The present multidisciplinary study aimed to examine the health behavior of Austrian college and university students, differentiated based on diet types (vegan, vegetarian, and omnivorous) and physical activity (PA) habits.

**Methods:**

Following a cross-sectional study design, a total number of 6,148 students (65.3% females; 66.1% bachelor students, 67.0% from urban areas; mean age: 24.8 years) from 52 Austrian college/universities participated in an online survey and provided data on sociodemographic characteristics, dietary patterns, PA habits, and other lifestyle behavior characteristics, including alcohol intake and smoking.

**Results:**

Across the total sample, 74.0% had a normal weight (BMI = 18.5–25.0 kg/m^2^), while the prevalence of overweight/obesity (BMI ≥ 30.0 kg/m^2^) was lower in females than males and more in rural than urban students (*p* < 0.01). The general prevalence of vegetarian and vegan diets was 22.8 and 6.0%, respectively, with a predominance of females, graduates, and urban students compared to their peers (*p* < 0.01). The majority of students (79.3%) had a regular engagement in sport/exercise, with a predominance of vegetarian or vegan students compared to omnivores (*p* < 0.01). Vegans and vegetarians had a lower alcohol intake (*p* < 0.01) but no differences in smoking habits (*p* > 0.05) compared to omnivores. Students engaging in sport/exercise had a lower smoking rate and higher intake of fruits, vegetables, and fluids compared to inactive students (*p* < 0.01).

**Conclusion:**

The present findings suggest that diet type and PA habits of college/university students have an impact on other health behaviors, highlighting the interconnected nature of lifestyle habits and health behavior.

## 1. Introduction

Health is an essential component of any individual’s life. In fact, having good health allows a person to properly develop personally and socially, as well as to interact with others and effectively respond to external stimuli (1, 2). According to the United Nations Sustainable Development Goals (3) and the World Health Organization (WHO) Voluntary Global Targets on non-communicable diseases (NCDs) (4), health is among the major topics for human development and satisfaction. Sustainable health refers to the achievement of optimal health outcomes for individuals while also considering the public health impacts, specifically on the environment, society, and future generations (3, 5). Therefore, there is an overarching responsibility for health to include major contributions at all societal levels (6), which implies the urgency of health-oriented action competence and sustainable willingness to act (7). Despite the advancement of health knowledge, recent trends worldwide show a high prevalence of chronic health conditions, particularly NCDs (8–10), which were the cause of 74% of deaths worldwide (9).

Evidence indicates that health behavior plays a fundamental role in preventing and managing most chronic diseases, including obesity, cardiometabolic disorders (e.g., dyslipidemia, hyperglycemia, hypertension), and psychosocial problems (e.g., discrimination, social isolation, low self-esteem) (11–14). Although imperative at any stage of the life cycle, one’s health condition assumes an even higher value in specific periods of life in which an individual faces several changes that may affect behavior (15). One such period is, for instance, moving to a higher educational level (from primary to secondary education, from secondary to college, etc.) since, in many cases, this also implies changes in the social circle of peers, more complex school assignments, and even environmental changes (i.e., moving to new school facilities) (15). Among the behavioral variables that could offer a better insight into one’s health condition and make a balance between personal health and environmental/social sustainability, experts point out the importance of diet, physical activity (PA), and harmful habits such as tobacco or alcohol abuse (16).

Healthy diet plays a key role in sustainable lifestyle (17). One of the most appropriate dietary strategies to achieve sustainable lifelong health is adhering to a plant-based diet (18–20), commonly categorized as a vegetarian or vegan diet (21, 22). Global epidemiological data show that the prevalence of both vegan and vegetarian diets is increasing worldwide (23–25). It has been indicated that at least 10% of European adults adhere to a vegan or vegetarian diet (25, 26), and younger adults are more likely to follow plant-based diets than older adults (27–29). The health benefits of vegan and vegetarian diets, especially in the management of several chronic diseases, have also been noted by the Academy of Nutrition and Dietetics (30) and the Physicians Committee for Responsible Medicine (21). Evidence shows that compared to an omnivorous diet, plant-based diets may also lead to a healthier body mass index (BMI) (22, 31, 32) and a significant reduction in all-cause mortality (33–35). Regardless of diet type, however, assuring adequate consumption of healthy food options (e.g., fruits, vegetables, fluids, whole grains) are considered the basis of a healthy diet (36–39). To maximize health benefits, it has been suggested that a healthy diet should be complimentary to regular engagement in PA (40–43). To date, however, limited studies have investigated the associations between diet type, PA habits, and other health behaviors, particularly in educational settings (44, 45).

Regarding PA, several parameters are considered important indicators of an active lifestyle. One of the most commonly used indicators is the recommended amount of weekly PA (e.g., whether this is achieved or not) (46). In fact, according to several health-focused international entities, young adults should comply with at least 150 min of PA per week distributed over at least 3 days (47). According to the WHO (46, 47), this quantity is the minimum necessary for PA to benefit an individual’s health. Indeed, carrying out PA at or beyond the recommended time seems to be associated with several positive health effects (48, 49). Likewise, it is suggested that not achieving the minimum recommendation of PA may affect several health parameters, including worsening mental health (50). In addition, the type of PA carried out seems to be of relative importance. For instance, individuals may choose to be active in an unstructured way, unrelated to organized sport clubs or specific associations, or they can adhere and become members of entities and clubs offering structured sports/sporting activities. Unstructured PA has been associated with a high potential of positively affecting people’s health in different ways, including increased enjoyment and motivation to be active (51). The relationship between PA and health is highly significant regarding positive human development (52).

In addition to PA and diet, other lifestyle factors, such as the abuse of tobacco and alcohol, have been studied for decades in the field of behavioral sciences and health (53). Substance abuse may be considered of particular importance in young adults, taking into account the parallel social changes happening at this point in life (53). Tobacco abuse has been previously associated with several indicators of health, such as reduced attention and memory when combined with cannabis (54). Additionally, the Centers for Disease Control and Prevention (CDC) mentions that smoking may increase the risk of cancer, cardiovascular and lung diseases, diabetes, and other non-communicable illnesses (55). Regarding alcohol abuse, according to the WHO, it may cause more than 200 diseases and injuries (56). These outcomes are further confirmed in a study conducted by Lee and Lee (57), which found that males and females who binge drink had higher odds of experiencing depressive moods and suicidal intentions.

A critical life period is during the transfer from adolescence to adulthood, as not only purely biological changes occur but also psychological and social changes (58, 59). Research shows that health behavior and the associated consequences shape and develop at young ages and continue over the lifespan (60, 61), significantly impacting lifelong health and well-being (62). Educational settings (e.g., schools, colleges, universities) are of utmost importance in shaping and developing life-long health behavior (63, 64). In particular, the literature highly emphasizes the importance of monitoring and applying effective strategies to improve the health behavior of university students at the critical stage of young adulthood (65–67). Hence, health-related research at this stage and the observation of student health-related behavior can provide practitioners with invaluable information about preserving and improving their health status. Due to the sedentary nature of the academic routine, university students tend to exhibit a higher prevalence of sedentary behavior compared to the global average (68), with approximately 50% failing to meet the minimum recommended level of PA (69–71). They have also been found to be at a high risk of having an unbalanced diet (66). These findings, however, contradict the increasing trend of young adults adopting vegetarian and vegan diets (27–29), which emphasizes the need to evaluate their health behaviors respecting diet type differences.

Monitoring the current health-related habits of college/university students is the initial step towards planning and implementing practices that can help promote and maintain healthy conditions during such a decisive life period. Therefore, the aim of this study to investigate lifestyle behavior based on dietary subgroups (omnivorous, vegetarian, vegan) and analyze potential associations with PA patterns as well as sociodemographic characteristics (e.g., age, sex, BMI, and living environment) among Austrian college/university students. The conceptual framework of this study revolves around examining how university students’ lifestyle behaviors are impacted by their diet types, PA patterns, and sociodemographic characteristics. By analyzing these interactions, the study provides valuable insights into the complex nature of lifestyle behaviors and their potential underlying determinants among young adults.

## 2. Methods

### 2.1. Study design and participants

*Sustainably healthy—From Science 2 Highschool and University*^1^ is a cross-sectional study with a multidisciplinary approach using a multilevel cluster sampling strategy. As a seamless follow-up to the original school study^2^ (44, 72), the present nation-wide study was conducted on a large sample of Austrian college and university students. This study is supported by the Federal Ministry of Education, Science, and Research of Austria (BMBWF; Department 1/7—School and University Sports). The study protocol was approved by the ethics board of the associated tertiary educational entities; students from 52 of the total 102 Austrian colleges/universities (> 50%) participated. Further information is available in the study protocol (73).

The target population for participation in this study was all college and university students in Austria, which equals an eligible sample size of 376,050 students who were registered and attended an Austrian tertiary educational entity. The board of deans at every Austrian college/university was contacted and invited to participate with their students in the online survey with relevant information about the study aim, procedures, and instructions. At the closure of data collection (31. July 2021), a total number of 6,148 students (1.6% of the eligible sample size) completed the online survey.

### 2.2. Study procedures

**Figure 1:**
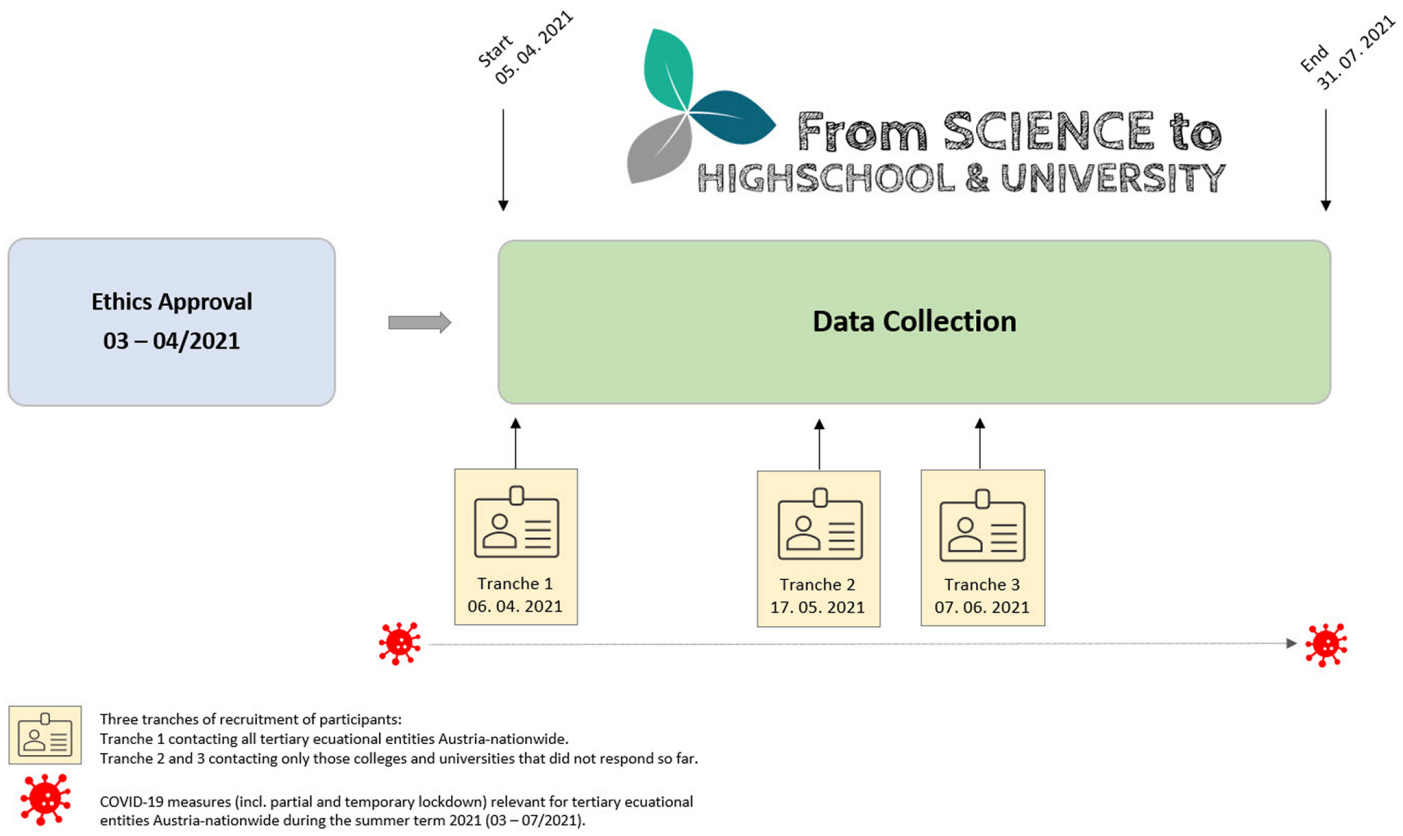
The study procedure and timescale from issuance of the ethics approval to the closure of data collection.

Data collection was open from the 5th of April through the 31st of July 2021. After getting familiar with the study objectives and procedures, students provided written informed consent to participate in this study. Participation was voluntary and anonymous, and students were able to withdraw at any time without providing reasons. Participants completed the standardized online questionnaire *via* an encrypted interface using a web link and could complete the survey comfortably within about 20 min by smartphone, tablet, or PC/laptop. Figure 1 shows the study procedure and timescale of the present study.

### 2.3. Measures and study variables

The online survey was provided in German and was based on self-report or self-assessment (LimeSurvey, version 3.25.15). The questionnaire consisted of six parts, including personal information (Part A), sports and exercise (Part B), nutrition (Part C), health and well-being (Part D), COVID-19 pandemic (Part E), and miscellaneous (Part F) (73). The survey was developed based on both the previous school study as its extension (72) as well as a comprehensive review of relevant literature, including validated questionnaires, large-scale scientific studies/reports, and recognized literature from renowned scientists, all of which are available in the study protocol (73). Participants were asked to respond to single-choice items, multiple-choice items, and choose and weigh their preferences among several options, which should be considered during data analysis and interpretation. However, considering the scope of the project (73), the present manuscript/study covers a general overview of the data, focusing on prevalences based on diet type and PA habits of participants.

In particular, the survey collected data on socio-demography (age, sex, nationality, federal state, living environment, marital status, highest academic level), anthropometry (height and body weight), study status (study level, subject area, current semester, university type), PA behavior (sports/exercise type, duration, frequency, participation in competitions, leisure-time activities, sports club membership, etc.), nutrition (current adherence to kind of diet, type and frequency of fluid intake, frequency of fruit and vegetable intake), and other lifestyle factors (amount and frequency of alcohol intake and smoking). Several control questions were included in the online questionnaire to identify contradictory information to increase the reliability of the data sets.

BMI was calculated using self-reported height and body weight (BW) values. Based on the WHO cut-points (74–76), participants were assigned to four BMI subgroups including underweight (≤ 18.5 kg/m^2^), normal-weight (18.5–25.0 kg/m^2^), overweight (25.0–30.0 kg/m^2^), and obese (≥ 30.0 kg/m^2^). Based on the self-reported diet types, participants were classified as vegetarian (those who do not eat meat, meat products, fish, and shellfish but consume dairy, eggs, and honey), vegan (no intake of any foods or ingredients from animal sources), or omnivorous diet (no dietary restriction on food sources) subgroups (30). For each type of sport/exercise engagement (i.e., leisure-time and sports club), participants were also categorized into those who engage in sport/exercise and those who do not. With regard to the frequency of sport/exercise (irrespective of the type of engagement), participants were assigned to three categories: “0–1 day/week,” “2–4 days/week,” and “5–7 days/week.” More detailed methodological information regarding the measuring tools and components can be find in the study protocol (73) as well as on the projects’ website (See footnote 1).

### 2.4. Data clearance

**Figure 2:**
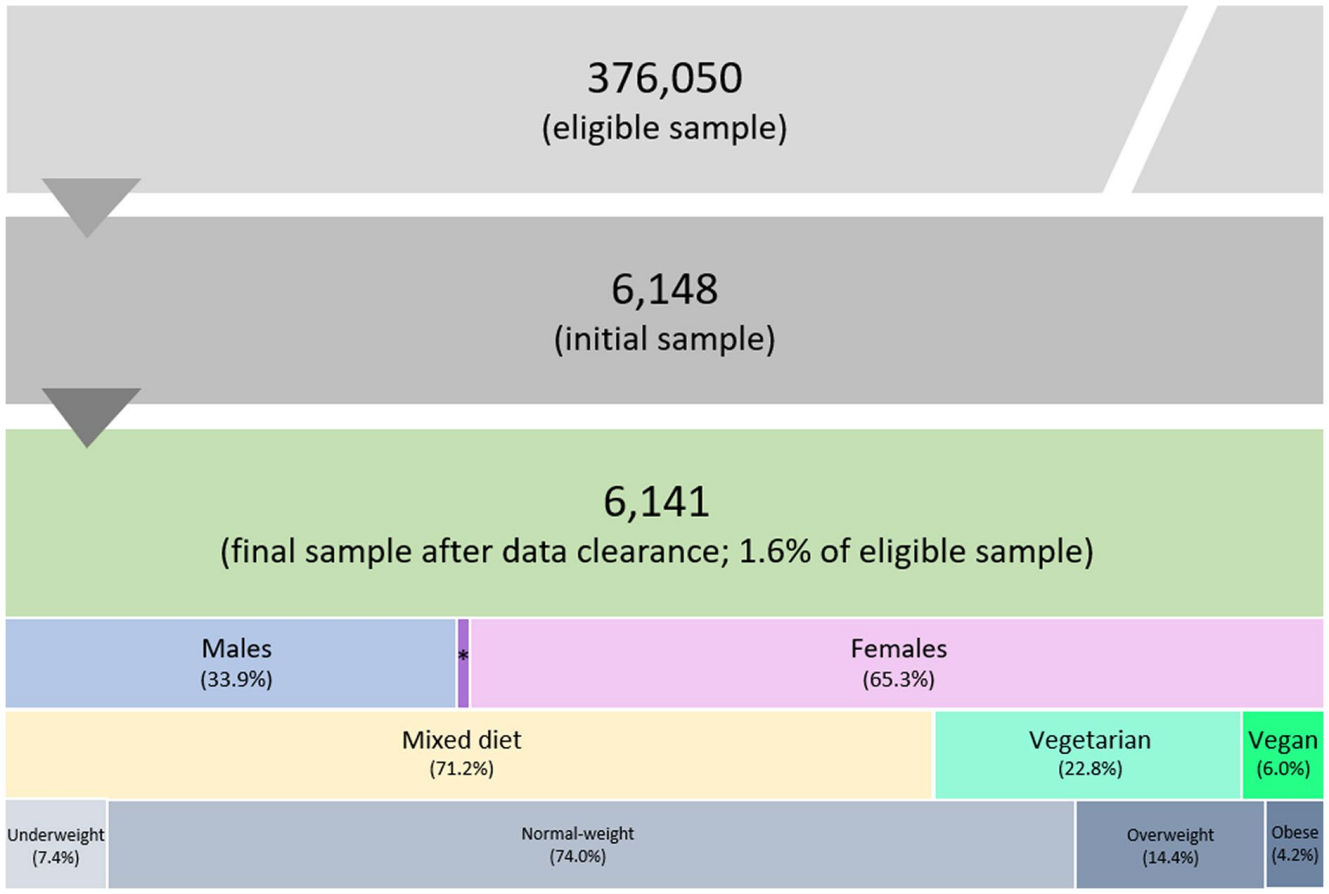
Flow chart of the sample size and classification of students based on sex, diet type, and BMI categories by WHO (59–61). ^*^Diverse population, representing 0.7% of the final sample size.

A total of 6,148 tertiary students completed the online survey. Participants with an age of less than 17 years as well as those with implausible or missing data for anthropometric characteristics were excluded from the study sample, which resulted in a final sample size of 6,141 (33.9% male, 65.3% female, 0.7% diverse) from all 9 federal states of Austria. Figure 2 shows the sample size and classification of participants.

### 2.5. Statistical analysis

All statistical tests were performed using SPSS 26.0 (SPSS Inc., IBM Corp., Armonk, NY, United States). Exploratory analysis was performed by descriptive statistics, and data are reported as mean with standard deviation (± SD; for continuous data) or prevalence/percentage (for nominal data). Multivariate analysis of variance (MANOVA) was used to examine differences in anthropometric characteristics and age by sex, study level, PA behavior, and diet type. Chi-square tests were conducted to examine differences in sex, study level, living environment, nationality, and health behavior by sports participation and diet type. The statistical significance level was set at *p* ≤ 0.05.

## 3. Results

66.1% of the final sample were bachelor students, while 33.9% were graduate students (master or doctorate). Across the entire sample, 67.0% lived in urban areas with the largest number of participants attending a university in Vienna (*n* = 3,012). 80.4% of students were Austrian. The most common nationalities among non-Austrian participants were German (9.0%) and Italian (4.6%) while other nationalities (*n* = 57) represented less than 1% of the study population. The distribution of participants by living environment, nationality, and level of study is shown in Table 1.

**Table 1:** Sample distribution by sex and study level presented as number of participants (n) and prevalence (%).

		Total (*n*)	Male *n* (%)	Female *n* (%)	Diverse *n* (%)	Bachelors *n* (%)	Graduates *n* (%)
		6,141	2,082 (33.9)	4,014 (65.3)	45 (0.7)	3,780 (61.6)	2,361 (38.4)
Living environment							
	Urban	4,113	1,555 (37.8)	2,522 (61.3)	36 (0.9)	2,251 (54.7)	1,862 (45.3)
	Rural	2,028	527 (26.0)	1,492 (73.6)	9 (0.4)	1,529 (75.4)	499 (24.6)
Nationality							
	Austrian	4,935	1,661 (33.7)	3,240 (65.7)	34 (0.7)	3,175 (64.3)	1,760 (35.7)
	Other	1,206	421 (34.9)	774 (64.2)	11 (0.9)	605 (50.2)	601 (49.8)
Federal State of Institution							
Burgenland		138	32 (23.2)	106 (76.8)	0 (0.0)	113 (81.9)	25 (18.1)
Carinthia		44	8 (18.2)	36 (81.8)	0 (0.0)	31 (70.5)	13 (29.5)
Lower Austria		329	91 (27.7)	237 (72.0)	1 (0.3)	255 (77.5)	74 (22.5)
Salzburg		178	32 (18.0)	145 (81.5)	1 (0.6)	107 (60.1)	71 (39.9)
Styria		458	123 (26.9)	335 (73.1)	0 (0.0)	321 (70.1)	137 (29.9)
Tyrol		1,298	354 (27.3)	938 (72.3)	6 (0.5)	811 (62.5)	487 (37.5)
Upper Austria		606	153 (25.2)	451 (74.4)	2 (0.3)	473 (78.1)	133 (21.9)
Vienna		3,012	1,277 (42.4)	1700 (56.4)	35 (1.2)	1,608 (53.4)	1,404 (46.6)
Vorarlberg		78	12 (15.4)	66 (84.6)	0 (0.0)	61 (78.2)	17 (21.8)

### 3.1. Anthropometric characteristics

Anthropometric characteristics across the total sample as well as by sex and student level (bachelor vs. graduate student) are shown in Table 2. Supplementary data (Supplementary Tables A1, A2; see Appendix) show additional anthropometric data by federal state and study level (separately for urban and rural areas). BW differed significantly between all sex groups (*p* < 0.01). Male participants were significantly taller than females and those reporting diverse sex (*p* < 0.01), while females had a significantly lower BMI than the other groups (*p* < 0.01). Accordingly, the prevalence of overweight and obesity was significantly lower in females (14.9%) compared to the male (25.6%) and diverse (35.6%) participants (*p* < 0.01). Further, bachelor students had a lower BMI than graduate students (*p* < 0.01) but there was no difference in the prevalence of overweight/obesity. The prevalence of overweight and obesity was higher in participants living in rural areas compared to those living urban areas (20.3 vs. 17.7%; *p* < 0.01).

**Table 2:** Anthropometric characteristics of the total sample, by sex as well as for bachelor and graduate students are presented as mean ± SD and prevalence for body weight categories.

	Total	Male	Female	Diverse	Bachelors	Graduates
Age (years)^1^^,^^2^	24.8 ± 6.3	25.6 ± 6.6	24.4 ± 6.0	26.8 ± 7.9	23.4 ± 5.8	27.1 ± 6.3
Urban	25.1 ± 6.0	25.7 ± 6.3	24.7 ± 5.8	26.4 ± 6.7	23.5 ± 5.6	27.1 ± 5.8
Rural	24.2 ± 6.8	25.3 ± 7.5	23.7 ± 6.4	28.3 ± 11.9	23.2 ± 6.1	27.3 ± 7.7
Body Weight (kg)^1^^,^^3^	67.1 ± 14.0	77.4 ± 13.4	61.7 ± 11.0	68.4 ± 16.2	66.4 ± 13.9	68.2 ± 14.2
Urban	67.5 ± 14.2	77.1 ± 13.4	61.6 ± 11.0	69.4 ± 17.3	67.0 ± 14.0	68.2 ± 14.4
Rural	66.1 ± 13.7	78.1 ± 13.4	61.9 ± 11.1	64.6 ± 10.8	65.4 ± 13.6	68.3 ± 13.6
Height (cm)^1^^,^^3^^,^^4^	171.9 ± 9.3	181.1 ± 6.9	167.2 ± 6.2	168.2 ± 13.0	171.4 ± 9.1	172.7 ± 9.5
Urban	172.6 ± 9.5	181.2 ± 7.0	167.4 ± 6.4	168.7 ± 13.8	172.2 ± 9.2	173.0 ± 9.8
Rural	170.5 ± 8.8	180.8 ± 6.6	166.9 ± 5.9	166.4 ± 9.4	170.2 ± 8.7	171.5 ± 8.4
BMI (kg/m^2^)^4^	22.6 ± 3.7	23.6 ± 3.7	22.1 ± 3.6	24.1 ± 4.5	22.5 ± 3.7	22.8 ± 3.7
Urban	22.6 ± 3.7	23.5 ± 3.6	22.0 ± 3.6	24.2 ± 4.5	22.5 ± 3.7	22.7 ± 3.6
Rural	22.7 ± 3.8	23.9 ± 3.7	22.2 ± 3.7	23.5 ± 4.9	22.5 ± 3.7	23.2 ± 4.0
Underweight (%)	7.4	2.9	9.7	4.4	7.7	6.8
Urban	7.7	3.4	10.3	5.6	8.1	7.1
Rural	6.8	1.5	8.7	0.0	7.1	5.6
Normal weight (%)	74.0	72.5	75.4	60.0	74.3	73.4
Urban	74.5	72.5	75.9	58.3	74.5	74.5
Rural	72.9	68.5	74.5	66.7	74.0	69.5
Overweight (%)	14.4	20.7	11.0	26.7	13.6	15.7
Urban	14.0	19.6	10.3	27.8	13.1	15.0
Rural	15.3	23.9	12.3	22.2	14.4	18.2
Obese (%)^4^	4.2	4.9	3.9	8.9	4.4	4.0
Urban	3.7	4.4	3.4	8.3	4.3	3.3
Rural	5.0	6.1	4.6	11.1	4.4	6.6

1 Significant difference between students living in urban and rural areas across the total sample (*p* < 0.01).

2 Significant difference between female students living in urban and rural areas (*p* < 0.01).

3 Significant difference between bachelor students living in urban and rural areas (*p* < 0.01).

4 Significant difference between graduate students living in urban and rural areas (*p* < 0.01).

### 3.2. Sports/exercise participation

The distribution of sports and exercise engagement is displayed in Table 3, while Table 4 displays anthropometric characteristics by sports participation. 79.3% of students reported having regular sports/exercise participation during their leisure time, but 19.3% of the participants were active members in sports clubs. Across the entire sample, participants were engaged in sport/exercise with a frequency of 3.6 ± 1.5 days a week. There were no sex differences in leisure-time sports but more males than females were engaged in club sports (22.5 vs. 17.8%; *p* < 0.01). While there was no difference in club sports participation by study level, more graduate students reported engagement in leisure-time sports/exercise (*p* < 0.01). The frequency of weekly sports/exercise participation did not differ by study level. There were also no differences in leisure-time sports/exercise by the living environment but club sports participation was higher in students living in rural compared to urban areas (22.6 vs. 17.7%; *p* < 0.01). Similarly, there was no difference in leisure-time sports/exercise between Austrian and international students even though more Austrian students were engaged in club sports (*p* < 0.01). Only leisure-time sports/exercise participation was also associated with a lower BMI (*p* < 0.01), while both leisure-time sports/exercise and club sports were associated with a lower prevalence of overweight and obesity (*p* < 0.01).

**Table 3:** Sports participation by sex, study level, living environment, and nationality presented as number of participants (n) and prevalence (%), as well as means ± SD for number of days with sports per week.

	Leisure-time sports *n* (%)	Club sports n (%)	Sport days/week mean ± SD
Total sample	4,867 (79.3)	1,188 (19.3)	3.6 ± 1.5
Male	1,616 (77.6)	469 (22.5)	3.6 ± 1.6
Female	3,218 (80.2)	713 (17.8)	3.6 ± 1.5
Diverse	33 (73.3)	6 (13.3)	3.5 ± 1.5
*Study level*			
Bachelor Students	2,931 (77.5)	745 (19.7)	3.6 ± 1.5
Graduate Students	1,936 (82.0)	443 (18.8)	3.6 ± 1.5
*Living environment*			
Urban	3,247 (78.9)	730 (17.7)	3.6 ± 1.5
Rural	1,620 (79.9)	458 (22.6)	3.6 ± 1.5
Nationality			
Austria	3,937 (84.9)	1,007 (20.4)	3.6 ± 1.6
Other	930 (77.1)	181 (15.0)	3.5 ± 1.5

**Table 4:** Anthropometric characteristics by sports participation presented as means ± standard deviation (SD) and prevalence (%) for body weight categories.

	Leisure-time sports		Club sports	
	yes	no	yes	no
Age (years)^2^	24.8 ± 6.2	25.1 ± 6.8	24.4 ± 6.0	25.0 ± 6.4
Height (cm)^2^	171.9 ± 9.2	171.7 ± 9.1	173.0 ± 9.6	171.5 ± 9.1
Body weight (kg)^1^	66.6 ± 13.2	69.4 ± 17.6	67.5 ± 13.0	66.9 ± 14.3
BMI (kg/m^2^)^1^	22.4 ± 3.4	23.5 ± 5.1	22.4 ± 3.1	22.6 ± 3.9
Underweight (%)^1^^,^^2^	7.1	9.3	5.1	8.2
Normal weight (%)^1^^,^^2^	76.4	62.0	79.4	72.7
Overweight (%)^1^^,^^2^	13.4	19.0	12.5	14.6
Obese (%)^1^^,^^2^	3.1	9.7	3.1	4.5

1 Significant difference between sports participation during leisure time (*p* < 0.01).

2 Significant difference between club sports participation (*p* < 0.01).

### 3.3. Diet

The distribution of students based on diet type is displayed in Table 5 with Table 6 showing anthropometric characteristics by diet type. 71.2% of participants reported consuming an omnivorous diet. 22.8% of the students reported following a vegetarian and 6.0% a vegan diet. Participants with diverse sex reported an omnivorous diet less often than plant-based diets (*n* = 13 vs. *n* = 23, respectively). Accordingly, the kind of diet type differed significantly across sexes (females > males following vegetarian and vegan diets). Vegetarian and vegan diets were also more common among graduate students compared to bachelor students (31.0 vs. 27.4%; *p* < 0.01) and among urban compared to rural students (31.6 vs. 23.0%; *p* < 0.01). While there were no differences in the prevalence of a vegetarian diet between Austrian and international students, a vegan diet was more common in international compared to Austrian students (7.8 vs. 5.6%; *p* < 0.01). Participants reporting an omnivorous diet were significantly older than those reporting a vegetarian or vegan diet (*p* < 0.01). Further, participants reporting an omnivorous diet had a higher BMI (22.9 ± 3.9 kg/m^2^) than those reporting a vegetarian (21.8 ± 3.2 kg/m^2^) or vegan (21.6 ± 2.8 kg/m^2^) diet, which resulted in a significantly higher prevalence of overweight and obesity in participants reporting an omnivorous diet compared to those reporting a plant-based diet (*p* < 0.01).

**Table 5:** Diet type by sex, study level, living environment, and nationality presented as number of participants (*n*) and prevalence (%).

	Omnivorous n (%)	Vegetarian *n* (%)	Vegan *n* (%)
Total sample	**3,641 (71.2)**	**1,164 (22.8)**	**309 (6.0)**
Male	1,410 (82.1)	244 (14.2)	64 (3.7)
Female	2,218 (66.0)	907 (27.0)	235 (7.0)
Diverse	13 (36.1)	13 (36.1)	10 (27.8)
*Study level*			
Bachelor Students	2,261 (72.6)	672 (21.6)	180 (5.8)
Graduate Students	1,380 (69.0)	492 (24.6)	129 (6.4)
*Living environment*			
Urban	2,353 (68.4)	839 (24.4)	249 (7.2)
Rural	1,288 (77.0)	325 (19.4)	60 (3.6)
*Nationality*			
Austria	2,985 (71.9)	935 (22.5)	234 (5.6)
Other	656 (68.3)	229 (23.9)	75 (7.8)

**Table 6:** Anthropometric characteristics by diet type presented as means ± standard deviation (SD), as well as absolute (*n*) and relative (%) numbers of participants (*N*) and prevalence (%) for BMI categories.

	Omnivorous	Vegetarian	Vegan
Age (years)^1^^,^^2^	25.3 ± 6.9	24.0 ± 4.8	23.8 ± 4.0
Height (cm)^1^^,^^2^	172.7 ± 9.4	170.0 ± 8.4	169.4 ± 8.4
Body weight (kg)^1^^,^^2^	68.6 ± 14.6	63.2 ± 11.6	62.2 ± 10.0
BMI (kg/m^2^)^1^^,^^2^	22.9 ± 3.9	21.8 ± 3.2	21.6 ± 2.8
Underweight (%)^1^	229 (6.3)	116 (10.0)	32 (10.4)
Normal weight (%)^1^^,^^2^	2,631 (72.3)	924 (79.4)	250 (80.9)
Overweight (%)^1^^,^^2^	606 (16.6)	97 (8.3)	21 (6.8)
Obese (%)^1^^,^^2^	175 (4.8)	27 (2.3)	6 (1.9)

1 Significant difference between omnivorous diet and vegetarian diet (*p* < 0.01).

2 Significant difference between omnivorous diet and vegan diet (*p* < 0.01).

### 3.4. Health related behavior by sports and exercise and dietary intake

Table 7 displays the association between sports participation and health-related behavior. Sports participation, particularly during leisure time, was associated with a higher prevalence of daily fruit and vegetable consumption as well as fluid intake of more than 2 liters/day (*p* < 0.01). While being the most common choice, water was more often reported as the primary fluid consumed by students with regular leisure-time sport activities (*p* < 0.01). Similarly, the prevalence of daily fruit and vegetable intake, fluid intake above 2 liters/day, and reporting water as the most common fluid increased with a higher frequency of engagement in sports (*p* < 0.01). Smoking was also less common in students participating in sports, while alcohol consumption did not differ by participation in leisure time or club sports. Engaging in sports more often during the week was associated with lower alcohol consumption (*p* < 0.01).

**Table 7:** Health related behavior by sports participation presented as prevalence (%).

	Leisure-time sports		Club sports		Sport days/week		
	yes	no	yes	no	0–1 Days	2–4 Days	5–7 Days
Daily fruit intake^1^^,^^2^^,^^3^	61.2	35.0	61.6	56.0	39.0	58.0	71.9
Daily vegetable intake^1^^,^^3^	83.3	64.0	81.4	80.1	67.8	82.6	86.6
Fluid intake (> 2 L/day)^1^^,^^2^^,^^3^	43.3	28.7	49.0	38.9	28.9	39.6	56.0
Water as most common drink^1^^,^^3^	84.5	76.4	84.1	83.1	78.2	84.3	85.3
Alcohol intake^3^	72.6	68.2	74.0	71.4	70.4	74.9	66.4
Smoking^1^^,^^2^^,^^3^	10.8	18.0	8.6	12.8	16.4	11.9	8.0

1 Significant difference between sports participation during leisure time (*p* < 0.01).

2 Significant difference between club sports participation (*p* < 0.01).

3 Significant difference between sport days/week (*p* < 0.01).

Table 8 displays the association between diet type and health related behavior. Regular leisure-time sports participation was more common in participants reporting a vegetarian (88.7%) or vegan (92.6%) diet compared to omnivores (82.9%; *p* < 0.01), but there was no difference in club sports participation across dietary subgroups. Accordingly, the number of days students participated in sports differed significantly across dietary groups (*p* < 0.01), with participants reporting a vegan diet displaying the highest number of days per week with sports participation, and those reporting an omnivorous diet displaying the lowest frequency of sports participation (vegan diet: 3.8 ± 1.8 days/week; vegetarian diet: 3.3 ± 1.9 days/week; omnivorous diet 2.9 ± 1.9 days/week).

**Table 8:** Health related behavior by kind of diet as prevalence (%).

	Omnivorous	Vegetarian	Vegan
Leisure-time sports participation^1^^,^^2^	82.9	88.7	92.6
Club sports participation	22.1	22.7	20.1
Fluid intake > 2 L/day	41.1	40.0	46.2
Water as most common drink^1^	82.0	86.2	86.7
Alcohol intake^1^^,^^2^	73.3	69.3	67.5
Smoking	12.3	10.5	11.7

1 Significant difference between omnivorous diet and vegetarian diet (*p* < 0.01).

2 Significant difference between omnivorous diet and vegan diet (*p* < 0.01).

While there was no difference in the total fluid intake across dietary subgroups, students with a vegetarian diet more often reported water as the primary drink compared to those who followed an omnivorous diet (*p* < 0.01). No difference was observed for smoking behavior across dietary subgroups, while vegetarian and vegan living students reported a lower alcohol consumption than participants with an omnivorous diet (*p* < 0.04). Additional information on sports participation, eating behavior, alcohol consumption, and smoking by federal states and living environment is provided in the Appendix as Supplementary Tables A3 and A4.

## 4. Discussion

The present Austria nationwide study aimed to examine health-related behavior of college and university students focusing on the prevalence of vegan, vegetarian, and omnivorous diets linked to sports & exercise patterns. This study is the first to investigate the dual approach of sustainable health (sports and exercise related to diet) based on a large and representative sample of a total of 6,141 students of 52 Austrian colleges/universities associated with sociodemographic factors (including sex, age, BMI, nationality, living environment) as well as other lifestyle factors (including alcohol intake and smoking). The most considerable findings are: (i) 3 out of 4 students (74.0%) were found to have a normal BMI that corresponds to a healthy BW; (ii) overweight/obesity was less prevalent in females (14.9%) than males (25.6%) and in rural students than those who live in urban areas; (iii) the majority of students (71.2%) reported to follow an omnivorous diet, while the prevalence of vegetarian and vegan diets were 22.8 and 6.0%, respectively; (iv) plant-based diets were more prevalent among females than males, graduate rather than bachelor students, and urban rather than rural students; (v) the majority of students (79.3%) reported regular sports engagement during their leisure time but only 1 out of 5 (19.3%) were active members of sports clubs; (vi) regular participation in leisure-time sports and exercise was more common in students reporting a vegetarian or vegan diet, while there was no difference in club sports participation across dietary subgroups; (vii) no difference in smoking behavior was found between diet types but students on plant-based diets reported a lower intake of alcohol; (viii) students who are engaged in sports and exercise activities reported having a lower smoking rate and healthier dietary behavior (in terms of fruits, vegetables, and fluid intake) compared to inactive students.

College and university students are in a critical period of life for adopting and stabilizing sustainable and lifelong healthy behavior (59, 60). The importance of this period is not limited to age-related characteristics (being in emerging adulthood); university life is independently associated with psychological stressors that undesirably affect health and future life (77). Data from a previous study demonstrated that 60% of university students do not meet the minimum PA recommendations and 47% have an unbalanced diet (66). Compared to these findings, college/university students in the present study had generally healthier behaviors with regard to sport/exercise engagement and dietary habits. In addition, the low prevalence of obesity and overweight in our sample (18.6%) corroborates the existing evidence from the WHO European Regional Obesity Report 2022, in which the Austrian population present among the lowest rates of combined obesity and overweight in Europe, and also globally (78). Moreover, this is in line with the Organization for Economic Cooperation and Development (OECD) report on the health status in the Austrian population, with about 17% of adults allocated in the category of overweight/obesity (79). Considering the strong relationship between physical inactivity and obesity rates (80), our data seem to be confirmed by both the existing evidence regarding adults’ PA levels and the data collected in the present research. Particularly, our study points out that more than 3 of every 4 college students are physically active, regardless of the type of sport/exercise chosen (unstructured or as a part of a membership in sports clubs). While it should be noted that the PA assessment method used in the present study does not provide a clear determination of the percentage of participants who met the minimum PA recommendations, the present findings seem to be consistent with the results from another study reporting that Austrian adults are among the most physically active in Europe, with three-quarters of them meeting the PA recommendations (79). Similarly, a total of around 75% of Austrian adults are reported to achieve the recommended weekly PA, according to data retrieved by the WHO (81). Indeed, higher PA leads to a more active metabolism and greater energy expenditure, which in turn increases the ability of the body to lose body weight or maintain it within healthy ranges (82). Previous studies have additionally tested different types of exercise (modality, intensity), finding that all of them have a positive effect on obesity and overweight, although with moderate differences from each other (83). Although the present cross-sectional study is unable to provide information on the effects of leisure-time sport/exercise and club sports on body weight, data show that the prevalence of excess body weight was lower in physically active students (especially those with leisure-time sport/exercise engagement) compared to non-active students. Health organizations, such as the CDC, confirmed that PA has a direct effect on weight loss as well as several indirect consequences, such as improved blood pressure, increased metabolism, or better cardiovascular health (84).

A deeper analysis of our findings also shows that most of the sport/exercise carried out by the participants of our study is provided by leisure-time, unstructured sport/exercise (79.3%) rather than membership in sports clubs (19.3%). While current guidelines do not clearly specify whether leisure-time sport/exercise or sports club activities may have a higher priority for health outcomes (46, 47), evidence indicates that both types can significantly promote overall health and well-being (85, 86). However, each of these types of PA may have advantages and disadvantages towards active behavior maintenance. For instance, both may be affected by time availability, as reported by Koh et al. (87). Time management may significantly change when attending college/university due to a new studying schedule (as classes may be distributed throughout the day, whereas they tend to be concentrated in shorter intervals and typically in the morning at lower education levels) as well as several changes in the social environment and general lifestyle (15). Furthermore, both types of PA may be influenced by environmental characteristics, such as the availability of sports facilities (88) or landscape features (mountain, beach, etc.) (89). Again, several college and university students often have to relocate to attend the new school, which may cause rearrangements and changes in their previous sport/exercise schedules to fit the new environment. Nonetheless, a major point separating leisure-time sport and exercise carried out in sports clubs may be that the latter requires the payment of a membership fee and, consequently, is affected by the individual’s financial availability. Previous studies have highlighted that willingness to pay for a membership depends, among other factors, on personal income (90, 91). Since college and university students usually leave their legal guardian household and become independent, they also need to autonomously sustain their life expenses, potentially driving them towards free-of-cost solutions for their daily PA. The positive effect of PA on weight control seems to be higher when combined with a proper diet (92), and the present data appears to confirm this statement. As mentioned above, nutritional behavior was also found to be positive in our sample, especially in those students who are also sufficiently active. On the one side, previous data suggest that increased PA may have a positive influence on individual choice of healthier nutritional habits (93). On the other side, combining a healthy diet and sufficient PA produces higher odds of improved health conditions than PA or nutrition alone (94). In particular, Elliot and Hamin found that nutrition and PA together provided 17.5 times higher chances of losing weight than PA alone (5.2 times) or diet only (7.2 times) in the observed sample (94).

In the present study, the prevalence of vegetarian/vegan diets were 28.8%, with a higher rate in females, graduates, and urban students compared to male, bachelor, and rural students, respectively. The higher prevalence of plant-based diets in the present study may be partially justified by the reports indicating younger adults are more interested in following plant-based diets (27–29) or the greater prevalence in German-speaking countries compared to the European average (29, 95, 96). Vegan and vegetarian diets seem to have the larger relationship with engagement in leisure-time sport/exercise in our sample. This finding is in line with previous reports indicating vegetarians and vegans may have healthier lifestyle habits, including a higher involvement in PA, sport, and exercise (44, 97). While the higher health-consciousness of individuals who follow a vegetarian/vegan diet compared to omnivores (98–100) may partially justify their higher engagement in sport/exercise, it should be considered that plant-based diets tend to be higher in complex carbohydrates, which provide sustained energy throughout the day (19, 30), and thus, may help them feel more energized and motivated to engage in sport/exercise. Although more research is required to explain the differences in behavioral patterns based on diet type, it is well known that vegetarian and vegan dietary patterns are equally adequate for providing individuals with all the essential nutrients beneficial for a healthy development (101) but also have a high protective effect against several diseases (21, 30), including cancer and diabetes. Therefore, along with sufficient PA, plant-based diets might provide extra benefits for individual and public health (102). Compared to omnivores, the rate of sport/exercise engagement in vegetarian and vegan students of the present study was 5.8 and 9.7% higher; however, students following an omnivorous diet also had a very high participation in leisure-time sport/exercise (82.9%). Regardless of personal preferences in diet type, the proper supply of fruits, vegetables, and whole grains and the consequential nutrient intake should be ensured in order to maximize the positive combined effect of diet and PA towards better weight management and, more generally, health status (36, 103).

Results from the present study show that the prevalence of alcohol intake in vegan and vegetarian students is lower than in omnivores, which is consistent with the previous findings (44, 104). While diet type had no association with the prevalence of smoking, it has also been found that smoking is less prevalent among physically active students compared to those with no regular sport/exercise. One possible justification may lie in the fact that physically active students may use sport/exercise as a way to relieve stress and tension rather than smoking since PA has been shown to release endorphins (known as natural mood-boosting chemicals) and can help reduce anxiety and stress (105). It has been well-documented that substance abuse is disadvantageous for the academic performance of university students (106, 107). However, a high prevalence of smoking and alcohol intake (40–70%) has been reported in college and university students (108–110), especially among male students (108, 111).

### 4.1. Limitations and strengths

Considering the cross-sectional design of the study in which the data were generated based on self-reports, there is a probability of socially desired statements, including underreporting (e.g., for body weight) and/or over-reporting (e.g., for PA habits). However, to maximize the reliability and validity of the data, several control questions were implemented in different parts of the survey with the purpose of identifying conflicting statements and minimizing the likelihood of misreporting. Accordingly, conflicting data were removed or revised from the associated analyses within the data clearance process. There might be confounding factors (including all direct and indirect lifestyle-related parameters), which may potentially affect the findings and the associated interpretations. We were also unable to validate the questionnaire since most variables in the survey were based on a single-item construct, which may originate from some pre-limitations such as lower precision in representing the related attribute, the low number of discrimination points, and the impossibility to assess internal consistency (112). However, it has been documented that single-item questionnaires may be considered valid (113), can be reliably used in the social sciences (114, 115), and may significantly reduce the limitations associated with lengthy surveys (116). Furthermore, it should be acknowledged that the method of PA assessment used in the study may not have accurately measured the prevalence of students who met PA recommendations. Therefore, caution should be considered when interpreting the findings related to the prevalence of meeting PA guidelines. Despite the existence of the abovementioned limitations, the present study has some strengths. This project has a multidisciplinary nature, including sport science, nutrition sciences, psychology, social sciences, and medicine. In addition, since current scientific evidence on the differentiated research questions is lacking on students in the tertiary educational setting, the present study is among the first to investigate the dual approach to sustainable health focusing on diet type and the associated epidemiological and sociodemographic aspects with a large sample.

### 4.2. Contribution to the field

Research evidence on health behavior provides a foundation for governments and health organizations to establish their policies and guidelines. Particularly, efforts towards enhancing the understanding of the interaction between diet type and lifestyle behavior during the early stages of adulthood is crucial for the promotion of public health. Therefore, the present findings provide a sound basis for reflecting on current health and lifestyle behavior (particularly the dual approach of sustainable health), which will ultimately help to reflect and update the current health-related recommendations. This study can also make a significant contribution to the development of an overview of the social trends in health behavior, especially in the respective populations in tertiary educational settings. Findings are expected to help empower college and university policies to design future offerings that consistently present healthy and sustainable choices of foods and meals across the environments (e.g., vending machines, canteen) accompanied by opportunities for PA, sport, and exercise. Alongside this, serious considerations should be applied in health-related educational policies to enhance health literacy and consequently improve the level of public health and well-being.

## 5. Conclusion

Despite the favorable health findings (concerning, e.g., BMI, prevalence of plant-based diets, regular engagement in sport/exercise), Austrian young adults had unsatisfactory health behavior regarding alcohol intake as well as fruit & vegetable consumption. Differentiated findings showed that vegan and vegetarian students and those with regular sport/exercise engagement show a slight tendency towards a healthier lifestyle. It can also be concluded that diet type and PA habits of college/university students have an impact on other health behaviors, highlighting the interconnected nature of lifestyle habits and health behavior. These data emphasize the necessity of continuing efforts to implement and facilitate healthy lifestyle choices for young Austrian populations. The present findings can be used by health specialists, decision-makers, and multipliers in health and education, and also may be considered a starting point for future interventions to improve individual and public health. However, the constant monitoring of health behavior should be a priority in health investigations to find and understand the longitudinal trajectories of health behavior, which have a lifelong and sustainable nature. Future research should be conducted to provide comparable data for a deeper understanding of the role of diet type and PA patterns on health behavior, health status, and physical performance of different healthy and unhealthy populations in sequential time points of life.

## Data availability statement

The datasets presented in this article are not readily available because the datasets presented in this article are not readily available because due to Austrian data security law, and additionally to the requirements of all the nine Austrian federal educational authorities considering data on pupils, it is not applicable. Requests to access the datasets should be directed to katharina.wirnitzer@ph-tirol.ac.at.

## Ethics statement

This study was conducted in accordance with the medical professional codex, the Helsinki Declaration as of 1996, Data Security Laws and good clinical practice guidelines. The study protocol was approved by both the ethics board of the Rectorate of the University College of Teacher Education Tyrol (PHT-Hsa-17-Z1.8-5n_4927; March 22, 2021) and the “Board for Ethical Questions in Science of the University of Innsbruck”, Vice-Rectorate for Research (Certificate of good standing, 22/2021; April 6, 2021). Informed consent was obtained from all participants involved in the study. Participation in the study was voluntary and could be terminated at any time without providing reason or negative consequences.

## Author contributions

KW, GR, WK, CD, and K-HW: conceptualization and study design. KW and CD: methodology and formal analysis. KW, CD, MM, and AC: writing original draft preparation. KW, MM, DT, MS, CD, GR, and WK: critical review and editing. GW: technical support. All authors contributed to the article and approved the submitted version.

## Funding

This Austria nationwide study is funded by the TWF (Tiroler Wissenschaftsförderung; reference number: F.30976/6-2021). However, the TWF was and is still not involved in the study procedures, and thus, there is and will be no impact from the funding agency on the study design, conduction and data collection, data analysis, presentations, and/or publication of the results. This Austria-wide college and university study is supported by the Austrian Federal Ministry of Education, Science and Research (BMBWF—Bundesministerium für Bildung, Wissenschaft und Forschung, Abteilung I/7—Schul-und Universitätssport), as well as by the Austrian Students’ Union (ÖH—Österreichische Hochschüler_innenschaft): https://uni.science2.school/en/#Partners.

## Conflict of interest

The authors declare that the research was conducted in the absence of any commercial or financial relationships that could be construed as a potential conflict of interest.

## Publisher’s note

All claims expressed in this article are solely those of the authors and do not necessarily represent those of their affiliated organizations, or those of the publisher, the editors and the reviewers. Any product that may be evaluated in this article, or claim that may be made by its manufacturer, is not guaranteed or endorsed by the publisher.

## Footnotes

^1^ https://uni.science2.school/en/

^2^ https://www.science2.school/en/
