# Supplementary material for: Health behavior of Austrian tertiary students focusing on diet type linked to sports and exercise—first glimpse of results from the “sustainably healthy—from science 2 high school and university” study
Source: Front Public Health. 2023 Jul 18;11:1129004. doi: 10.3389/fpubh.2023.1129004 (PMC10392833; doi:10.3389/fpubh.2023.1129004)
Supplement: Supplementary file 1 [file Table_1.pdf]

Appendix

Health Behaviors of Austrian Tertiary Students focusing on Diet Type linked to Sports & Exercise – First Glimpse of Results from the "Sustainably healthy – From Science 2 Highschool & University" Study

1 Supplementary Data

----

2 Supplementary Matrial: Tables A1 to A4

Table A1: Anthropometric Characteristics by federal state and living environment in bachelor students (n = 3780) presented as mean ± SD and prevalence (%) for the overweight/obesity category.

	N	Age (years)	Height (cm)	Body Weight (kg)	BMI (kg/m²)	Overweight/Obesity (%)
Burgenland	113	24.7 ± 7.5	169.8 ± 10.2	66.5 ± 15.8	22.9 ± 3.9	22.1
Urban	27	26.4 ±8.7	168.9 ± 9.7	65.0 ± 17.7	22.5 ± 4.3	18.5
Rural	86	24.2 ± 7.0	170.1 ± 10.4	67.0 ± 15.2	23.0 ± 3.9	23.3
Carinthia	31	28.6 ± 11.2	167.1 ± 11.2	65.2 ± 16.2	23.3 ± 4.5	22.6
Urban	18	26.8 ± 10.4	165.2 ± 12.2	65.2 ± 17.5	23.8 ± 5.4	22.2
Rural	13	31.0 ± 12.2	169.6 ± 9.6	65.2 ± 14.8	22.5 ± 3.1	23.1
Lower Austria	255	23.5 ± 6.5	170.5 ± 9.0	66.8 ± 15.0	22.9 ± 4.1	19.6
Urban	97	23.7 ± 6.1	171.0 ± 8.8	67.1 ± 15.1	22.9 ± 4.2	19.6
Rural	158	23.4 ± 6.7	170.2 ± 9.1	66.6 ± 14.9	22.9 ± 4.0	19.6
Salzburg	107	23.0 ± 4.6	168.2 ± 8.1	61.6 ± 11.4	21.7 ± 3.4	14.0
Urban	55	23.1 ± 4.9	168.8 ± 8.6	59.6 ± 9.0	20.9 ± 2.7	9.1
Rural	52	22.8 ± 4.4	167.5 ± 7.6	63.7 ± 13.2	22.6 ± 3.9	19.2
Styria	321	23.5 ± 6.1	170.2 ± 8.4	65.5 ± 12.9	22.5 ± 3.5	17.8
Urban	162	24.3 ± 7.2	171.0 ± 8.6	66.9 ± 13.6	22.8 ± 3.8	19.8
Rural	159	22.7 ± 4.6	169.3 ± 8.2	64.0 ± 12.0	22.2 ± 3.2	15.7
Tyrol	811	23.5 ± 6.2	170.4 ± 8.1	64.8 ± 12.7	22.2 ± 3.5	16.0
Urban	388	23.8 ± 6.2	171.3 ± 8.4	65.4 ± 12.8	22.2 ± 3.6	16.2
Rural	423	23.1 ± 6.2	169.5 ± 7.7	64.2 ± 12.6	22.2 ± 3.4	15.9
Upper Austria	473	24.0 ± 6.4	170.1 ± 8.8	65.2 ± 13.1	22.5 ± 3.6	17.5
Urban	217	24.2 ± 5.9	170.2 ± 8.7	64.8 ± 13.5	22.3 ± 3.7	15.2
Rural	256	23.8 ± 6.8	170.0 ± 8.8	65.6 ± 12.8	22.6 ± 3.6	19.5
Vienna	1607	22.9 ± 5.0	173.2 ± 9.4	68.0 ± 14.3	22.6 ± 3.8	18.7
Urban	1268	23.1 ± 4.9	173.4 ± 9.4	68.2 ± 14.2	22.6 ± 3.7	17.9
Rural	339	22.4 ± 5.1	172.3 ± 9.4	67.1 ± 14.7	22.5 ± 3.9	21.8
Vorarlberg	61	23.7 ± 5.5	168.9 ± 7.8	64.6 ± 16.2	22.5 ± 4.8	19.7
Urban	18	26.3 ± 7.1	171.5 ± 9.2	67.9 ± 17.9	22.9 ± 4.6	22.2
Rural	43	22.6 ± 4.3	167.7 ± 6.9	63.2 ± 15.5	22.4 ± 4.9	18.6

Table A2: Anthropometric Characteristics by federal state and living environment in graduate students (n = 2361) presented as mean ± SD and prevalence (%) for the overweight/obesity subsample.

	N	Age (years)	Height (cm)	Body Weight (kg)	BMI (kg/m²)	Overweight/Obesity (%)
Burgenland	25	27.7 ± 7.5	169.6 ± 7.9	65.6 ± 13.6	22.7 ± 3.6	24.0
Urban	12	26.5 ± 4.7	166.7 ± 8.2	60.2 ± 10.8	21.6 ± 3.3	16.7
Rural	13	28.8 ± 9.4	172.3 ± 6.9	70.7 ± 11.3	23.6 ± 3.7	30.8
Carinthia	13	28.2 ± 5.0	166.7 ± 8.3	66.6 ± 12.2	24.0 ± 3.9	38.5
Urban	7	30.4 ± 4.6	168.2 ± 9.2	70.1 ± 15.2	24.7 ± 4.7	42.9
Rural	6	25.7 ± 4.5	164.8 ± 7.5	62.5 ± 6.5	23.1 ± 2.8	33.3
Lower Austria	74	28.7 ± 7.8	171.9 ± 8.5	69.1 ± 13.5	23.4 ± 4.3	24.3
Urban	26	28.6 ± 7.5	172.4 ± 10.2	66.8 ± 12.8	22.3 ± 3.0	11.5
Rural	48	28.7 ± 8.0	171.6 ± 7.6	70.3 ± 13.8	23.9 ± 4.8	31.3
Salzburg	71	27.3 ± 6.6	173.2 ± 9.7	69.4 ± 17.6	22.9 ± 4.4	25.4
Urban	52	27.1 ± 5.5	173.5 ± 10.7	70.3 ± 19.3	23.1 ± 4.8	25.0
Rural	19	28.1 ± 9.0	172.5 ± 6.4	66.8 ± 12.1	22.3 ± 2.9	26.3
Styria	137	26.5 ± 5.6	173.3 ± 9.4	67.9 ± 12.0	22.5 ± 3.3	16.8
Urban	106	26.6 ± 5.8	173.5 ± 9.4	67.4 ± 12.0	22.3 ± 3.1	15.1
Rural	31	26.2 ± 5.3	172.5 ± 9.4	69.5 ± 12.0	23.4 ± 4.0	22.6
Tyrol	487	26.3 ± 6.2	171.6 ± 9.1	66.8 ± 12.9	22.6 ± 3.4	19.1
Urban	344	26.3 ± 5.7	171.9 ± 9.3	66.4 ± 113.4	22.4 ± 3.3	17.4
Rural	143	26.3 ± 7.4	171.0 ± 8.7	67.5 ± 11.8	23.0 ± 3.4	23.1
Upper Austria	133	30.0 ± 8.9	172.2 ± 9.4	68.4 ± 15.3	23.0 ± 4.0	18.8
Urban	79	29.1 ± 7.6	172.5 ± 9.9	68.4 ± 15.4	22.8 ± 3.7	15.2
Rural	54	31.3 ± 10.6	171.7 ± 8.8	68.5 ± 15.3	23.1 ± 4.7	24.1
Vienna	1404	27.0 ± 5.7	173.3 ± 9.7	68.7 ± 14.6	22.8 ± 3.7	19.6
Urban	1228	27.1 ± 5.5	173.5 ± 9.8	68.8 ± 14.6	22.7 ± 3.6	18.9
Rural	176	26.5 ± 6.6	171.9 ± 8.5	68.4 ± 14.8	23.1 ± 4.3	24.4
Vorarlberg	17	32.5 ± 13.1	169.7 ± 9.1	68.1 ± 14.1	23.6 ± 4.3	17.6
Urban	8	36.9 ± 16.8	172.0 ± 12.1	69.6 ± 10.5	23.5 ± 2.6	12.5
Rural	9	28.6 ± 7.8	167.7 ± 5.4	66.7 ± 17.3	23.7 ± 5.6	22.2

frontiers

Table A3: Health-related behaviors by federal state and living environment in bachelor students (n = 3,780) presented as prevalence (%) and mean ± SD for sport days per week

	N	Leisure time sports (%)	Club-sports (%)	Days/week with sport (mean ± SD)	Daily Fruit Intake (%)	Daily Vegetable Intake (%)	Fluid intake >2L/day (%)	Water most common fluid (%)	Vegetarian /Vegan (%)	Alcohol Intake (%)	Smoking (%)
Burgenland	113	85.0	17.6	3.1 ± 1.9	48.4	73.3	34.7	83.2	20.4	61.4	10.8
Urban	27	85.2	3.8	3.2 ± 1.8	50.0	75.0	37.5	75.0	20.8	59.1	9.1
Rural	86	85.0	22.4	3.0 ± 1.9	47.9	73.2	33.8	85.9	20.3	62.3	11.5
Carinthia	31	83.3	24.1	2.9 ± 1.8	59.3	70.4	29.6	77.8	17.9	68.2	18.2
Urban	18	88.2	25.0	3.3 ± 1.8	66.7	73.3	20.0	93.3	18.8	66.7	16.7
Rural	13	76.9	23.1	2.4 ± 1.7	50.0	66.7	41.7	58.3	16.7	70.0	20.0
Lower Austria	255	86.3	27.2	2.9 ± 1.8	52.5	74.8	31.7	81.7	20.0	73.5	10.5
Urban	97	90.1	33.7	2.9 ± 2.6	53.4	78.1	30.1	87.7	16.7	67.7	3.1
Rural	158	83.6	23.2	2.9 ± 1.9	51.9	72.9	32.6	78.3	22.0	76.7	14.7
Salzburg	107	85.6	23.3	2.9 ± 2.0	66.2	81.8	26.0	85.7	39.8	72.2	15.3
Urban	55	82.4	16.7	2.7 ± 1.9	63.4	80.5	24.4	87.8	43.5	73.0	18.9
Rural	52	89.1	31.0	3.1 ± 2.1	69.4	83.3	27.8	83.3	35.1	71.4	11.4
Styria	321	88.9	25.5	3.2 ± 1.8	59.1	78.7	40.2	84.3	28.5	73.1	7.6
Urban	162	87.5	24.3	3.2 ± 1.9	59.8	81.1	47.2	89.0	35.6	74.4	9.9
Rural	159	90.4	26.8	3.2 ± 1.8	58.3	76.4	33.1	79.5	21.1	71.8	5.1
Tyrol	811	87.5	24.2	3.2 ± 1.9	58.8	78.1	38.5	84.1	24.1	73.8	12.5
Urban	388	87.9	20.2	3.2 ± 1.9	59.1	81.4	42.5	86.4	29.4	78.1	14.9
Rural	423	87.2	28.0	3.1 ± 1.9	58.4	75.0	34.7	81.9	19.2	69.8	10.4
Upper Austria	473	83.0	24.8	3.0 ± 2.0	62.6	80.6	38.7	77.5	28.1	70.0	9.7
Urban	217	82.3	24.0	3.0 ± 2.0	60.2	84.9	39.2	79.5	35.3	67.9	12.8
Rural	256	83.6	25.5	2.9 ± 2.0	64.5	77.3	38.4	75.8	22.5	71.6	7.2
Vienna	1607	80.3	19.4	2.8 ± 2.0	52.8	78.4	42.7	83.7	30.3	70.9	13.9
Urban	1268	79.7	18.5	2.8 ± 2.0	52.1	78.4	43.3	83.5	30.7	73.2	15.2
Rural	339	82.4	22.8	3.1 ± 2.0	55.3	78.2	40.4	84.4	28.8	66.5	9.1
Vorarlberg	61	79.7	30.4	2.5 ± 1.8	57.7	75.0	11.5	75.0	9.3	80.9	19.1
Urban	18	76.5	35.3	2.9 ± 2.2	43.8	75.0	12.5	87.5	12.5	85.7	35.7
Rural	43	81.0	28.2	2.4 ± 1.8	63.9	75.0	11.1	69.4	7.9	78.8	12.1

Table A4: Health-related behaviors by federal state and living environment in graduate students (n = 2,361) presented as prevalence (%) and mean ± SD for sport days per week

	N	Leisure time sports (%)	Club-sports (%)	Days/week with sport (mean ± SD)	Daily Fruit Intake (%)	Daily Vegetable Intake (%)	Fluid intake >2L/day (%)	Water most common fluid (%)	Vegetarian /Vegan (%)	Alcohol Intake (%)	Smoking (%)
Burgenland	25	79.2	13.0	2.7 ± 1.9	61.9	100.0	42.9	76.2	19.2	73.7	5.3
Urban	12	81.8	10.0	2.5 ± 1.5	70.0	100.0	30.0	60.0	30.0	62.5	0.0
Rural	13	76.9	15.4	2.9 ± 2.2	54.5	100.0	54.5	90.9	8.3	81.8	9.1
Carinthia	13	90.9	36.4	3.6 ± 1.7	63.6	63.6	63.6	90.1	36.4	81.8	27.3
Urban	7	100.0	40.0	3.8 ± 1.6	60.0	60.0	60.0	100.0	40.0	80.0	40.0
Rural	6	83.3	33.3	3.5 ± 2.0	66.7	66.7	66.7	66.7	33.3	83.3	16.7
Lower Austria	74	88.4	27.3	3.0 ± 2.0	47.6	79.4	46.0	76.2	26.6	61.4	8.8
Urban	26	87.0	21.7	2.9 ± 1.9	43.5	87.0	39.1	69.6	26.1	54.5	18.2
Rural	48	89.1	30.2	3.1 ± 2.1	50.0	75.0	50.0	80.0	26.8	65.7	2.9
Salzburg	71	93.9	23.1	3.2 ± 1.8	67.7	90.3	37.1	90.3	27.0	61.5	11.5
Urban	52	91.4	23.4	3.2 ± 2.0	66.7	88.9	37.7	91.1	30.4	61.0	7.3
Rural	19	100.0	22.2	3.0 ± 1.2	70.6	94.1	35.3	88.2	17.6	63.6	27.3
Styria	137	91.5	22.4	3.5 ± 1.8	59.6	87.2	49.5	84.4	33.9	68.3	7.7
Urban	106	92.9	21.1	3.6 ± 1.8	58.3	89.3	56.0	85.7	34.1	70.0	7.5
Rural	31	87.1	26.7	3.2 ± 1.8	64.0	80.0	28.0	80.0	33.3	62.5	8.3
Tyrol	487	91.7	23.8	3.4 ± 1.9	61.7	85.5	40.1	84.2	31.1	73.8	9.8
Urban	344	92.1	20.8	3.4 ± 1.8	62.0	88.2	43.3	86.1	35.3	74.3	9.8
Rural	143	90.7	31.2	3.3 ± 2.1	60.7	78.6	32.1	79.5	21.4	72.5	9.8
Upper Austria	133	93.1	22.3	3.0 ± 1.7	60.0	82.9	47.6	80.0	26.4	69.8	5.2
Urban	79	92.5	24.6	3.0 ± 1.8	43.5	80.6	50.0	83.9	30.6	76.8	3.6
Rural	54	100.0	19.1	3.0 ± 1.6	65.1	86.0	44.2	74.4	20.5	60.0	75
Vienna	1404	86.1	19.3	3.0 ± 1.9	58.2	83.3	45.3	84.6	32.1	73.4	12.4
Urban	1228	86.3	18.8	3.0 ± 1.9	57.8	84.3	46.1	84.9	32.7	74.1	13.2
Rural	176	84.2	22.9	2.8 ± 2.0	61.2	75.5	39.6	82.0	27.9	71.5	6.9
Vorarlberg	17	87.5	26.7	3.0 ± 1.9	42.9	57.1	35.7	92.9	7.1	76.9	15.4
Urban	8	71.4	0.0	1.8 ± 1.6	0.0	20.0	20.0	100.0	0.0	100.0	25.0
Rural	9	100.0	44.4	3.8 ± 1.6	66.7	77.8	44.4	88.9	11.1	66.7	11.1
